# Supplementary material for: Feasibility of a Culturally Specific DEmentia Competence Education for Nursing Home Taskforce (DECENT) Programme: A Mixed-Method Approach
Source: Int J Environ Res Public Health. 2022 Dec 12;19(24):16679. doi: 10.3390/ijerph192416679 (PMC9779405; doi:10.3390/ijerph192416679)
Supplement: Supplementary file 1 [file ijerph-19-16679-s001.zip › ijerph-2034646-supplementary.pdf]

Table S1: Outline of the DECENT programme

Topic	Objective	Content	Competencies in framework	Format
1. Understanding dementia and dementia related issues	· To describe the pathophysiological changes of dementia	· Definition and common misunderstanding of dementia<br>· Most common types of dementia<br>· Different stages of dementia<br>· Common signs and symptoms of dementia<br>· Normal ageing and dementia<br>· Risk factors and preventative strategies of dementia<br>· The losses and impact associated with a diagnosis of dementia and increasing losses as the condition processes<br>· The differences between dementia, depression and delirium<br>· Introduction of end-of-life care for people with dementia	· Knowledge and understanding of dementia<br>· Preventative strategies and health promotion<br>· End-of-life care	· Lecture<br>· Discussion<br>· Handouts
2. Person-centred care	· To introduce the concept and principles of person-centred care	· The concept of person-centred care<br>· The principles and values of person-centred care<br>· Main points of implementing person-centred care<br>· Approaches suggested to promote person-centred care in daily care for residents with dementia by taking the local context into consideration	· Person-centred care<br>· Physical and psychological well-being of people with dementia<br>· Ethics in dementia care<br>· Teamwork	· Lecture<br>· Case study<br>· Discussion<br>· Reflection<br>· Handouts
3. Care communication	· To identify factors that influence communication<br>· To demonstrate skills for communicating with people with dementia	· Factors that commonly affect the communication with residents with dementia in Chinese nursing home settings<br>· Impact of communication on residents with dementia<br>· Verbal and non-verbal communication skills	· Communication;<br>· Physical and psychological well-being of people with dementia;<br>· Teamwork	· Lecture<br>· Case study<br>· Discussion<br>· Refection<br>· Video clips

		· How to find possible communication topics with residents with dementia<br>· Other communication tips for resident with dementia		· Handouts
4. Understanding challenging behaviours and management skills	· To identify reasons for challenging behaviours<br>· To describe effective strategies for dealing with challenging behaviours	· The potential reasons that may trigger challenging behaviours of residents with dementia in nursing-home settings<br>· How to understand their behaviours<br>· The tool helping assess residents' challenging behaviours<br>· How to manage challenging behaviours	· Understanding of the related challenging behaviours<br>· Management strategies<br>· Physical and psychological well-being of people with dementia<br>· Ethics in dementia care	· Lecture<br>· Case study<br>· Discussion<br>· Reflection<br>· Video clips<br>· Handouts
5. Dementia care in daily living activities	· To describe ways for dealing with common difficulties in activities of daily living, such as eating, toilet and sleeping	· The overall principles of caring people with dementia with their daily living activities<br>· Most concerned safety issues Specific care skills in commonly daily living activities, including diet, urination and defecation, shower, sleep and daily activities	· Daily living activities;<br>· Physical and psychological well-being of people with dementia;<br>· Ethics in dementia care	· Lecture<br>· Case study<br>· Discussion<br>· Reflection<br>· Video clips<br>· Handouts
6. Dementia-friendly environment	· To introduce the impact of environment on people with dementia<br>· To introduce the principles of dementia-friendly environment	· The impact of nursing home environment on residents with dementia<br>· Principles for designing commonly used room and areas in nursing homes, including the bathroom, outside of bathroom, corridor, bedroom, dining room, living room and entrance	· Environment;<br>· Physical and psychological well-being of people with dementia	· Lecture<br>· Case study<br>· Discussion<br>· Reflection<br>· Handouts
7. Interaction with families	· To identify the importance of interacting with families<br>· To introduce the strategies for interacting with families	· Knowing and understanding family caregivers<br>· Providing support for family caregivers<br>· Cooperation with family caregivers in caring residents with dementia, especially in the transition period from home to nursing home setting<br>· Strategies to strengthen understanding between family caregivers and staff	· Interaction with families	· Lecture<br>· Discussion<br>· Reflection<br>· Handouts

8. Care staff's self-care and development	· To introduce the importance of self-care<br>· To identify ways for self-care and personal development	· Commonly encountered problems and causes identified from the field visits and interviews with staff in nursing homes and literatures<br>· Suggested strategies to assess and coping with these problems from the perspective of individuals, cooperating with others and suggestions for work development	· Care staff's self-care and development;<br>· Teamwork	· Lecture<br>· Discussion<br>· Reflection<br>· Handouts

DECENT: DEmentia Competence Education for Nursing home Taskforce

Table S2:

Revised Standards for Quality Improvement Reporting Excellence
SQUIRE 2.0

Notes to Authors		Page
Title and Abstract		
**1. Title**	Indicate that the manuscript concerns an initiative to improve healthcare (broadly defined to include the quality, safety, effectiveness, patient-centeredness, timeliness, cost, efficiency, and equity of healthcare)	1
**2. Abstract**	a. Provide adequate information to aid in searching and indexing<br>b. Summarize all key information from various sections of the text using the abstract format of the intended publication or a structured summary such as: background, local problem, methods, interventions, results, conclusions	1-2
Introduction	*Why did you start?*	
**3. Problem Description**	Nature and significance of the local problem	5-6
**4. Available Knowledge**	Summary of what is currently known about the problem, including relevant previous studies	4-5
**5. Rationale**	Informal or formal frameworks, models, concepts, and/or theories used to explain the problem, any reasons or assumptions that were used to develop the intervention(s), and reasons why the intervention(s) was expected to work	4-6
**6. Specific Aims**	Purpose of the project and of this report	7
Methods	*What did you do?*	
**7. Context**	Contextual elements considered important at the outset of introducing the intervention(s)	6
**8. Intervention(s)**	a. Description of the intervention(s) in sufficient detail that others could reproduce it<br>b. Specifics of the team involved in the work	6, 7-9
**9. Study of the Intervention(s)**	a. Approach chosen for assessing the impact of the intervention(s)<br>b. Approach used to establish whether the observed outcomes were due to the intervention(s)	9-10
**10. Measures**	a. Measures chosen for studying processes and outcomes of the intervention(s), including rationale for choosing them, their operational definitions, and their validity and reliability<br>b. Description of the approach to the ongoing assessment of contextual elements that contributed to the success, failure, efficiency, and cost	9-10

	c. Methods employed for assessing completeness and accuracy of data	
**11. Analysis**	a. Qualitative and quantitative methods used to draw inferences from the data<br>b. Methods for understanding variation within the data, including the effects of time as a variable	11-12
**12. Ethical Considerations**	Ethical aspects of implementing and studying the intervention(s) and how they were addressed, including, but not limited to, formal ethics review and potential conflict(s) of interest	11
Results	*What did you find?*	
**13. Results**	a. Initial steps of the intervention(s) and their evolution over time (e.g., time-line diagram, flow chart, or table), including modifications made to the intervention during the project<br>b. Details of the process measures and outcome<br>c. Contextual elements that interacted with the intervention(s)<br>d. Observed associations between outcomes, interventions, and relevant contextual elements<br>e. Unintended consequences such as unexpected benefits, problems, failures, or costs associated with the intervention(s).<br>f. Details about missing data	12-16
Discussion	*What does it mean?*	
**14. Summary**	a. Key findings, including relevance to the rationale and specific aims<br>b. Particular strengths of the project	17
**15. Interpretation**	a. Nature of the association between the intervention(s) and the outcomes<br>b. Comparison of results with findings from other publications<br>c. Impact of the project on people and systems<br>d. Reasons for any differences between observed and anticipated outcomes, including the influence of context<br>e. Costs and strategic trade-offs, including opportunity costs	17-18
**16. Limitations**	a. Limits to the generalizability of the work<br>b. Factors that might have limited internal validity such as confounding, bias, or imprecision in the design, methods, measurement, or analysis<br>c. Efforts made to minimize and adjust for limitations	18
**17. Conclusions**	a. Usefulness of the work<br>b. Sustainability<br>c. Potential for spread to other contexts<br>d. Implications for practice and for further study in the field<br>e. Suggested next steps	19

Other Information		
**18. Funding**	Sources of funding that supported this work. Role, if any, of the funding organization in the design, implementation, interpretation, and reporting	20
